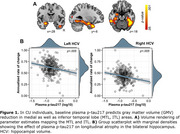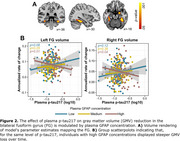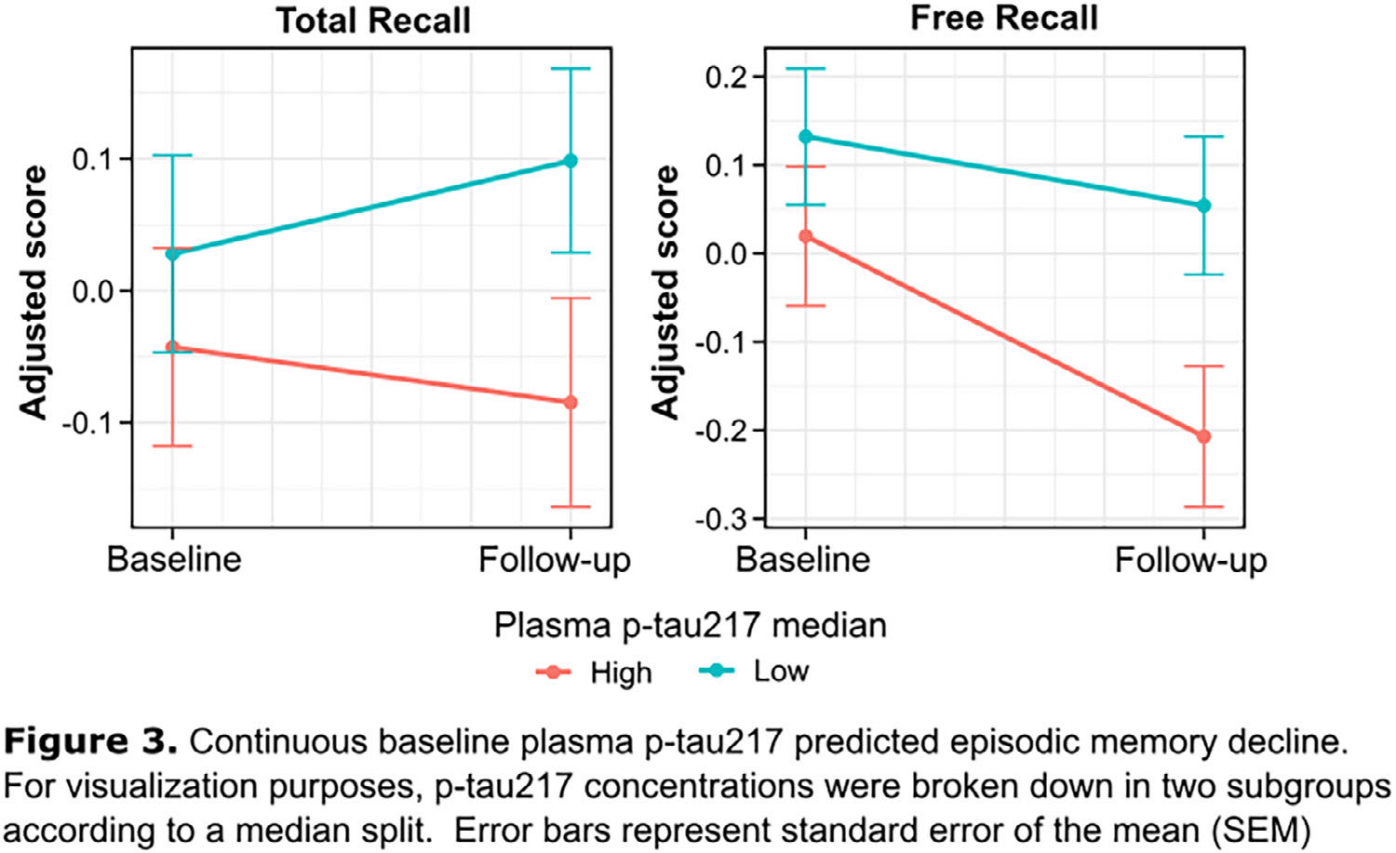# Synergistic Effects of Plasma *p*‐tau217 and GFAP on Neurodegeneration and Cognitive Decline in Cognitively Unimpaired Individuals

**DOI:** 10.1002/alz70856_098969

**Published:** 2025-12-24

**Authors:** Raffaele Cacciaglia, Armand González Escalante, Paula Ortiz‐Romero, Oriol Grau‐Rivera, Gonzalo Sánchez‐Benavides, Theresa A. Day, Kaj Blennow, Henrik Zetterberg, Jose Luis Molinuevo, Juan Domingo Gispert, Marc Suárez‐Calvet, Gemma Salvadó

**Affiliations:** ^1^ Barcelonaβeta Brain Research Center (BBRC), Pasqual Maragall Foundation, Barcelona, Spain; ^2^ Centro de Investigación Biomédica en Red de Fragilidad y Envejecimiento Saludable (CIBERFES), Instituto de Salud Carlos III, Madrid, Spain; ^3^ IMIM (Hospital del Mar Medical Research Institute), Barcelona, Spain; ^4^ Servei de Neurologia, Hospital del Mar, Barcelona, Spain; ^5^ Lilly Research Laboratories, Eli Lilly and Company, Indianapolis, IN, USA; ^6^ Clinical Neurochemistry Laboratory, Sahlgrenska University Hospital, Mölndal, Sweden; ^7^ Department of Psychiatry and Neurochemistry, Institute of Neuroscience & Physiology, the Sahlgrenska Academy at the University of Gothenburg, Mölndal, Gothenburg, Sweden; ^8^ Hong Kong Center for Neurodegenerative Diseases, Hong Kong, Science Park, China; ^9^ Department of Neurodegenerative Disease, UCL Institute of Neurology, Queen Square, London, United Kingdom; ^10^ UK Dementia Research Institute, University College London, London, United Kingdom; ^11^ Clinical Neurochemistry Laboratory, Sahlgrenska University Hospital, Gothenburg, Sweden; ^12^ School of Medicine & Public Health, University of Wisconsin‐Madison, Madison, WI, USA; ^13^ Department of Psychiatry and Neurochemistry, Institute of Neuroscience and Physiology, The Sahlgrenska Academy, University of Gothenburg, Mölndal, Sweden; ^14^ Lundbeck A/S, Copenhagen, Denmark; ^15^ AstraZeneca, Barcelona, Spain; ^16^ CIBER Bioingeniería, Biomateriales y Nanomedicina (CIBER‐BBN), Madrid, Spain; ^17^ Pompeu Fabra University, Barcelona, Spain; ^18^ Clinical Memory Research Unit, Department of Clinical Sciences Malmö, Lund University, Lund, Sweden

## Abstract

**Background:**

Plasma phosphorylated tau at threonine 217 (*p*‐tau217) is an accurate biomarker for Alzheimer's pathology. Emerging data suggest that plasma *p*‐tau217, especially when combined with Glial Fibrillary Acidic Protein (GFAP), predicts cognitive decline up to a decade before clinical onset. However, no studies have quantified its impact on both gray matter volume (GMV) atrophy and cognitive decline in CU individuals.

**Method:**

We included 329 CU individuals from the ALFA+ study (mean age = 60.86; SD = 4.75) with baseline fluid biomarkers, longitudinal MRI and cognitive data (mean follow‐up: 3.35 years; SD = 0.53). The impact of plasma *p*‐tau217 on GMV changes and episodic memory (EM) decline, assessed via the Free and Cued Selective Reminding Test (FCSRT), was examined. Plasma GFAP and neurofilament light chain (NfL) were quantified using the GFAP Discovery and NF‐light Advantage commercial kits, respectively. Plasma Ab42/40 was measured with the commercial Neurology 4‐Plex E Advantage Kit, and plasma *p*‐tau217 with the MesoScale Discovery platform (MSD) by Eli Lilly and Company. Brain atrophy was measured using SPM longitudinal registration, with results significant at *p* < 0.005 (cluster extent: 100 voxels). EM decline was modeled with linear mixed‐effects models with random intercepts, adjusted for baseline age, sex, education, *APOE*‐ε4, Aβ42/40, and NfL. Plasma *p*‐tau217 and GFAP interaction effects were also tested.

**Result:**

Baseline plasma *p*‐tau217 concentrations were significantly associated with GMV atrophy in the hippocampus, inferior temporal and fusiform gyrus, bilaterally (Figure 1A‐B). A significant interaction was observed, indicating that, for a given level of plasma *p*‐tau217, individuals with elevated plasma GFAP showed greater GMV loss (Figure 2A‐B). Finally, plasma *p*‐tau217 significantly predicted EM decline in the total recall (TR) and free recall (FR) of the FCSRT (Figure 3), while no significant interactions were found with GFAP.

**Conclusion:**

Plasma *p*‐tau217 is associated with longitudinal neurodegeneration and cognitive decline even in CU individuals. The observed interaction with plasma GFAP may suggest an early role of astroglial reactivity in neurodegeneration, particularly in medial temporal lobe regions. These findings underscore the utility of plasma *p*‐tau217 as a diagnostic tool and for stratifying high‐risk individuals in preclinical Alzheimer's.